# Implementation science in times of Covid-19

**DOI:** 10.1186/s13012-020-01006-x

**Published:** 2020-06-08

**Authors:** Michel Wensing, Anne Sales, Rebecca Armstrong, Paul Wilson

**Affiliations:** 1 Department of General Practice and Health Services Research, University Hospital Heidelberg, Heidelberg, Germany; 2 Faculty of Medicine, University of Heidelberg, Heidelberg, Germany; 3 Department of Veteran Affairs Center for Clinical Management Research, VA Ann Arbor Healthcare System, Ann Arbor, MI USA; 4 Department of Learning Health Sciences, University of Michigan Medical School, Ann Arbor, MI USA; 5 National Disability Insurance Agency, Melbourne, Australia; 6 University of Manchester, Manchester, UK; 7 NIHR Applied Research Collaboration Greater Manchester, Manchester, UK

## Abstract

The emergence of SARS-CoV-2/Covid-19 affects all of us and is associated with rapid and massive changes in healthcare and societies. As a response, a range of interventions for patients and populations have been implemented in health and preventive settings, or need to be implemented in the short and long term. Implementation science offers a multidisciplinary perspective and systematic approach for the design, evaluation and analysis of programmes and policies to enhance implementation. The emergence of Covid-19 provides an urgent need to develop new perspectives and approaches in implementation science, such as the addition of innovative and rigorous approaches to the collection, use and analysis of ‘real-world’ data. Above all, we hope that implementation scientists will focus on what they can contribute to manage Covid-19 and its consequences for people, healthcare and society.

## Backgrounds

SARS-CoV-2/Covid-19 (further: Covid-19) has affected many of us, including loved ones, colleagues, clinicians and most particularly vulnerable people such as the elderly and people with chronic disease. The emergence of Covid-19 is associated with major changes in human behaviours, institutions and societies, compressed in time and replicated rapidly throughout the globe. Research evidence to guide the direction of these changes is quickly evolving, yet decision-makers face major uncertainties. The sustainability of the changes, given the lack of infrastructure to support them, is questionable. This is made more challenging by the growing realization of their huge negative economic impacts. The health sector has been turbulent in most societies for many years, but this up-ending of the sector creates chaotic conditions that merge both behaviour change and economic uncertainties, for a threatening environment. The extent of the chaos brings some seeds of opportunity, and implementation science may be primed to act in the current and future environments.

Covid-19 has had unquestionable impact on societies and specifically on healthcare across the world. Dedicated facilities for diagnosis and triage of patients with suspected Covid-19 have been established in primary care-, ambulatory- and community-based settings. Treatment and care of patients with Covid-19 has been organized in hospitals and ambulatory settings, followed by the rehabilitation of patients after a stay in intensive care units, thus affecting every sector of the healthcare system. Measures for the prevention of infections in the population have been intensified through many recommendations and regulations regarding hygiene and protection for airborne infections. Systems for early detection and tracking of infected individuals in the population are being set up in many countries. A set of preventive measures described as ‘social distancing’, while beneficial in reducing the spread of the virus, is causing new health problems (e.g. mental health problems, lifestyle-related diseases, family and domestic violence) that will need increased attention of healthcare providers in the coming period. While there is much hope for an effective vaccine, this would need to be provided to the entire global population within the shortest possible time on a scale that is unprecedented.

Furthermore, the overwhelming attention on Covid-19 has also impacted healthcare provision for patients with other diseases, some of which are also acutely life-threatening, leading to delayed use of essential medical examinations and treatments. News media report that hospitals have been very quiet in recent times and that the negative impact of changes in non-Covid healthcare on population health is higher than the direct impact of Covid-19 [1]. This seems to be caused both by a lowered attendance of healthcare visits by patients and by the decreased access to specialist care, such as diagnostic testing after screening for cancers, due to decreased capacities. These impacts on non-Covid healthcare are believed to be a major driver of excess deaths, which can be observed in the weekly total mortality rates [2].

From an implementation science perspective, the current situation presents a unique set of circumstances. The amount of immediate information on Covid-19 is very high: there is an ongoing flow of research evidence (much of it not yet peer reviewed, or minimally reviewed [3]), clinical guidance, regulations by authorities and messages in the media. In many countries, the numbers of hospital admissions and deaths due to Covid-19 are reported daily in the general media. Much of this information is uncertain, inconsistent and quickly replaced or complemented by new insights and guidance [4]. Also, the information does not always apply locally, because of differences in infection rates, testing regimes, availability of medical resources and social and geographic factors (e.g. population density). We also observe that many decision-makers in times of Covid-19 are prepared to take radical decisions. This is almost opposite to previous situations, when many decision-makers were not particularly inclined to implement new practices. Arguably, the current times with Covid-19 have also led to higher trust in health professionals, scientific researchers, public health organizations and public authorities, although this trend does not apply across the board.

We believe that implementation science has increasing relevance in the currently evolving later phases of the pandemic, when the expanding research evidence is starting to consolidate. Also, evidence regarding similar viruses and similar diseases can be extrapolated regarding some aspects of prevention, treatment and recovery. At present, the need exists to emphasize awareness and primacy of the strength of the evidence as prioritization decisions are made, for at least three reasons: first, to ensure that the dictum ‘First, do no harm’ is maintained, and implementation efforts focus on effective practices that will improve, not harm, health; second, to optimize the clinical effectiveness of treatment and care provided in routine practice; and third, because perception of evidence is often a critical factor in determining implementation success. The debates that have roiled around claims of effective treatment or approaches to control Covid-19 provide good examples of the latter issue, and similarly, the question of possible harm has been a central factor in mainstream press and social media coverage. As the evidence on prevention and treatment of Covid-19 is quickly expanding, we anticipate that the role of implementation science will quickly grow in the coming period.

## Implementation challenges

Implementation science offers a multidisciplinary perspective and systematic approach for the design, evaluation and analysis of implementation initiatives. Implementation science can provide recommendations for the implementation of a specific practice, or stop an outdated practice, in a given setting and target group. To provide such recommendations, we need empirical research of the implementation problems and implementation strategies in these settings and target groups. While it rarely provides recommendations that guarantee successful implementation with absolute certainty, it can increase success in implementing practices. Implementation scientists often function as a reminder that implementation needs attention, that it is never sufficient to produce and disseminate information for changing behaviour, organizations and systems. This will become more important when the effects of Covid-19 last longer than a short period of weeks or months.

As a response to Covid-19, a range of interventions for patients and populations have been implemented in health and preventive settings, or need to be implemented, including:

- Preventive interventions to reduce the transmission of SARS-CoV-2 in the population and reduce the rates of Covid-19;
- Preventive interventions to reduce transmission among healthcare workers to avoid nosocomial infections in healthcare institutions;
- Organizational changes to separate patients with (suspected) infection, such as separate sites, clinical teams or times for these patients, and use of information technologies to deliver healthcare;
- Procedures for diagnosis and triage in patients with suspected infection, including screening and advance care planning;
- Procedures for treatment and care in patients with Covid-19, including rehabilitation after intensive treatment;
- Treatment and care of health problems that are caused by some of the preventive interventions, for instance in mental healthcare;
- Treatment and reorganization of care of other diseases and health conditions, for which essential treatment was reduced or delayed due to Covid-19.

Some of these interventions have been implemented quickly and comprehensively, using well-established approaches to make changes in routine practice. The changes in intensive care units, which have included rapid construction of new negative pressure rooms and massive increases in critical care admissions, may provide an example of this. Maintaining newly implemented practices, however, poses challenges, if they were instituted quickly without adequate infrastructures (e.g. sufficient nurses trained for intensive care, sufficient numbers of respiratory therapists to safely manage mechanical ventilation). Also, given the uncertainties in the research evidence, the guidance and regulations on these interventions are likely to change over time. For instance, separate ambulatory practices for testing and triaging patients with suspected COVID-19 may no longer be needed if local infection rates are low. The implementation of recommended practices may get more challenging, when specific measures are softened. We observe that the degree and type of softening varies between geographic regions, which is likely to complicate the implementation of population health interventions. For instance, currently prevailing local regulations for physical movement vary widely within the USA.

For other interventions, the actual implementation in practice has been challenging from the start. For instance, remote care through telemedicine (or through telephone) continues to meet with barriers in Germany. The shortage of masks has made it difficult to reduce the spread of infections by healthcare workers in nursing homes in the Netherlands. Shortage of supplies and poor geographic distribution of critical inventory, such as ventilators and renal replacement technologies, have resulted in suboptimal care in areas of the USA. The UK has seen significant shortcomings in equipment procurement at the national level [5], albeit a much stronger local response particularly in relation to data sharing and rapid reconfigurations of acute and primary care services. Countries with relatively low rates of infections and deaths, such as Australia, may not take all required measures to prepare for a situation of rising infection rates.

We have identified a number of areas that seem crucial for implementation science beyond the immediate response to Covid-19, and we would welcome submissions to *Implementation Science* and *Implementation Science Communications* on those:

- Implementation of interventions to reduce the transmission of airborne infections in healthcare settings, such as hand hygiene and use of protective masks, using behavioural change frameworks [6];
- Implementation of telehealth, including consultations by telephone, building on available frameworks and research evidence [7–9];
- Scale-up and sustainability of interventions for managing Covid-19, an area that needed development before Covid-19 [10];
- Mitigating adverse impacts of interventions for Covid-19, such as inequalities in access to healthcare, or inequitable treatment for vulnerable populations [11];
- Mitigation of adverse impacts on non-Covid healthcare, such as delayed use of essential medical screening and treatment.

## New perspectives on implementation science

The emergence of Covid-19 provides an urgent need to develop new perspectives and approaches in implementation science, which contribute to its development in the longer run. For instance, there is a need for further development and application of rapid methods of research synthesis [12]. Traditional ‘normal’ science approaches to knowledge synthesis are likely to be deemed inadequate and irrelevant in crisis conditions. One phenomenon that is emerging in the crisis atmosphere is the rapid cycling from new idea to considerable excitement, and then rapid ‘bursting the bubble’ when the evidence does not support the idea on initial testing. A critical challenge is to synthesize rapidly, with the best evidence possible. New robust approaches to evidence synthesis (e.g. rapid 48-h reviews, crowd-sourcing for evidence reviews, living reviews) will be needed.

Furthermore, the emergence of Covid-19 allows the analysis of change under conditions that are fundamentally different from what constituted usual care until recently. For instance, the coordination of patient care seems to have partly shifted (back) to a hierarchical mode for the management of Covid-19. In settings that are heavily under pressure, such as intensive care units, collaborative decision-making in team meetings has often been replaced by single-person leadership and reduction of face-to-face interactions to what is absolutely necessary. Will this hierarchical mode have lasting impact, also in other domains of healthcare? How will this change moderate the effectiveness of implementation strategies, particularly those that depend on teamwork and critical reflection on individual routines?

Another example of an important perspective on implementation science in the context of Covid-19 concerns the role of social networks. Following theories on diffusion of innovations, implementation science has recognized early that social networks are also crucial for the uptake of innovations [13, 14]. Healthcare providers who are embedded in large networks tend be more exposed to innovations and, if they are sufficiently dense, more inclined to adopt these. Social networks are also relevant for the contagion of the infectious diseases. What impact do social distancing measures have in the longer run on the uptake of innovations, considering the lowered rotation in clinical teams, the reduced number of participants at handovers in patient care and the limitations imposed on at-home healthcare? These and other research questions need to be developed and answered in future research. We would be interested to receive studies that examine implementation under these ‘post-Covid-19’ conditions.

We recognize that many studies may need to make changes to how or when data are collected to reduce the burden on clinical services and/or to the reduce risk of transmission. *Implementation Science* has long advocated prospective quantitative and qualitative data collection through experimental and quasi-experimental designs. The current climate necessitates more use of data that are collected routinely for clinical or administrative purposes. We therefore welcome innovative and rigorous approaches to the collection, use and analysis of ‘real-world’ data. This may include retrospective designs such as large-scale comparative case studies within or between countries, although we will continue to judge these on a case-by-case basis.

## Conclusion

These times of Covid-19 are highly worrying as well as highly fascinating. Echoing previous calls [15], above all, we hope that implementation scientists will not fall in love with the problems, and focus on what they can contribute to manage the disease and its consequences for people, healthcare and society.
